# Analysis of the variability among radiation oncologists in delineation of the postsurgical tumor bed based on 4D-CT

**DOI:** 10.18632/oncotarget.12044

**Published:** 2016-09-15

**Authors:** Wei Wang, Jianbin Li, Jun Xing, Min Xu, Qian Shao, Tingyong Fan, Bing Guo, Shanshan Liu

**Affiliations:** ^1^ Department of Radiation Oncology, Shandong Cancer Hospital affiliated to Shandong University, Shandong Academy of Medical Sciences, Jinan, Shandong province, China, 250117

**Keywords:** breast-conserving treatment, tumor bed delineation, surgical clip number, CT slice thickness, cavity visualization score

## Abstract

**Objective:**

This study investigated interobserver and intraobserver variability in radiation oncologists' definition of the tumor bed (TB) after breast-conserving surgery (BCS).

**Results:**

The TB volume, CVS and number of surgical clips were not significantly related to intraobserver variability. Moreover, no correlation was noted between CT slice thickness and interobserver variability (Δ_inter_, DSC_inter_) in TB delineation, and no significant difference was noted among the three groups. The TB volume was negatively correlated with Δ_inter_. DSC_inter_ improved significantly with increased TB volume and decreased Δ_inter_. DSC_inter_ also increased significantly in patients with a CVS of 3 to 5 compared with patients with a CVS of 1 to 2. DSC_inter_ was thus positively correlated with the CVS, with a correlation coefficient of 0.451. The use of 7 to 9 surgical clips neither decreased Δ_inter_ nor increased DSC_inter._

**Materials and Methods:**

Five or more surgical clips were placed at the TB during lumpectomy. The TB was delineated on the end expiration scan. The data were stratified based on the cavity visualization score (CVS), CT slice thickness and surgical clip number. The Dice similarity coefficient (DSC) and inter(intra)observer variability (Δ_inter_ and Δ_intra_) in different groups were evaluated and compared.

**Conclusions:**

Inter(intra)observer variability in TB delineation was decreased for breast cancer patients implanted with 5 or more surgical clips in the cohort with a higher CVS and a larger TB. The use of more than 6 surgical clips did not significantly improve TB delineation, so 5 to 6 surgical clips are likely adequate to delineate the TB.

## INTRODUCTION

Developments in radiation therapy delivery after breast-conserving surgery (BCS), such as three-dimensional conformal external beam radiotherapy (3D-CRT), intensity-modulated radiotherapy (IMRT), and image-guided radiation therapy (IGRT), have improved the accuracy of radiation delivery [1–3]. Whole-breast irradiation (WBI) with or without a tumor bed (TB) boost in combination with BCS remains the standard treatment for early-stage breast cancer. However, external beam partial breast irradiation (EB-PBI) has recently attracted more attention in this field [4–6]. Several issues and unanswered questions remain regarding the use of EB-PBI or WBI with a TB boost after BCS, including inter-fraction breathing motion and intra-fraction set-up variation, which may lead to displacement and deformation of the planning target volume (PTV). Definite delineation and contouring of the TB are also crucially important. Many factors influence TB identification, such as the cavity visualization score (CVS) on computed tomography (CT), the interval from BCS to the CT simulation scan, the number of surgical clips placed during surgery, and breast distortion after surgery [5–9].

Accurate localization and delineation of the TB are most critical in patients undergoing WBI with a TB boost and EB-PBI. However, the definition of the TB volume after BCS is controversial [7, 8, 10–13], and no consensus is available regarding the first-choice modality for TB contouring. Three-dimensional CT (3D-CT) is broadly applied in radiotherapy simulation, and the TB is delineated on 3D-CT images. However, 3D-CT does not avoid the effects of breathing motion on the accuracy of target volume delineation. The image-guided deep inspiration breath hold (DIBH) treatment protocol is a feasible irradiation method with small setup variability that significantly reduces the dose to the heart [14–16]. However, the accuracy of active breathing control (ABC) is altered by pulmonary function and the threshold of inspiration capacity that is chosen to perform DIBH. Four-dimensional CT (4D-CT) has also been evaluated in a few planning studies for breast cancer radiotherapy simulation, and it significantly reduces the target delineation errors caused by motion artifacts [17–19].

Additionally, recent studies demonstrated that 4D-CT-based target delineation was more accurate for WBI and EB-PBI [20, 21]. Delineation standard setting, delineation training, the CVS, and the number of surgical clips are all very important for the accuracy of TB delineation [7, 22–24]. In particular, recent studies suggested that the placement of 4 to 6 surgical clips may improve the accuracy of target structure delineation [5, 11, 25]. However, whether further increases in the number of surgical clips within the TB will improve the accuracy of EB-PBI with a TB boost following or concurrent with WBI delivery is not known. The effects of different CT slice thicknesses, the TB volume, and the CVS on the concordance among radiation oncologists in TB determination when 5 or more surgical clips are placed at the TB are also not clear. Here, by evaluating the relationship between the number of clips and seroma-based TB delineation on postoperative 4D-CT scans for EB-PBI and boost following or concurrent with WBI delivery, we investigated whether the presence of more surgical clips would improve interobserver and intraobserver variability in radiation oncologists' accuracy in TB delineation.

## RESULTS

### Patients and treatment

A total of 66 patients with SIB or EB-PBI were enrolled in this study. Seventeen patients refused undergo 4D-CT simulation, fourteen patients were excluded because they were receiving neoadjuvant chemotherapy for a tumor larger than 5 cm. Therefore, 35 early-stage breast cancer patients who had undergone BCS were eligible. Table 1 outlines the patient characteristics. The mean patient age was 43.0 years (range 21 to 60 years). Eighteen of the 35 patients had right-sided breast cancer, and the remaining 17 patients had left-sided breast cancer.

**Table 1:** Clinical and TB characteristics of 35 patients

Variable	Value
Mean age (y)	43.0
Minimum	21
Maximum	60
T stage	
Tis	9
T1	19
T2	7
Histology	
DCIS	9
IDC	21
Other	5
Tumor location	
Left-sided	17
Right-sided	18
TB size (cm^3^)	25.2 ± 15.0
Range	4.59-79.3
CVS	2.51 ± 1.50
CVS-1	13
CVS-2	7
CVS-3	4
CVS-4	6
CVS-5	5
No. of clips	6.31 ± 1.23
5	12
6	8
7	9
8	4
9	2

Abbreviations: TB = tumor bed; CVS = cavity visualization score; DCIS = ductal carcinoma in situ; IDC = invasive ductal carcinoma.

### Influence of CT slice thickness on TB delineation

The 4D-CT scans of the 35 patients with early breast cancer who had undergone BCS constituted the dataset for this retrospective study. The 4D-CT images for fourteen patients were reconstructed using a thickness of 2 mm, and 3-mm and 5-mm thicknesses were used for 14, 14 and 7 patients, respectively. Δ_intra_, Δ_inter_, DSC_intra_, and DSC_inter_ for all patients were 7.78 ± 5.99%, 27.2 ± 25.5%, 87.0 ± 4.47% and 73.8 ± 9.31%, respectively. The patients were divided into three groups based on CT thickness. Statistical analyses demonstrated no significant differences in Δ_intra_, Δ_inter_, DSC_intra_, or DSC_inter_ among the three groups (F = 0.472, 1.636, 0.799, and 0.322, respectively; P = 0.628, 0.211, 0.459, and 0.727, respectively). CT slice thickness thus did not influence Δ_intra_, Δ_inter_, DSC_intra_, or DSC_inter_ (r = 0.161, 0.143, 0.207, and 0.093, respectively).

### TB delineation analysis

We also investigated variations in TB definition using various factors that may influence TB delineation, including the TB volume, CVS, and presence of surgical clips (Table 2). The mean volume of the TB was 25.2 ± 15.0 cm^3^. The mean Δ_inter_ for the TB was reduced to 15.6%, from 37.0% (P = 0.009), and the mean DSC_inter_ for the TB increased to 77.6%, from 70.6% (P = 0.025), with the increase in the TB volume. A significant inverse correlation was thus identified for the TB volume and interobserver variability in TB delineation.

**Table 2:** Factors influencing the delineation of the TB

Influencing factor	No. of patients	Δ_intra_(%)	Δ_inter_(%)	DSC_intra_(%)	DSC_inter_(%)
V_mean_ (cm^3^)					
<25.2	19	7.14	37.0	86.8	70.6
≥ 25.2	16	8.54	15.6	87.2	77.6
*T*		−0.664	2.796	−0.245	−2.343
P		0.512	0.009	0.808	0.025
Correction (r)	35	−0.019	−0.485	0.297	0.510
CVS					
1-2	20	7.96	32.2	86.3	70.1
3-5	15	7.54	20.6	87.9	78.8
*T*		0.201	1.433	−0.141	−3.051
P		0.842	0.161	0.305	0.004
Correction (r)	35	−0.019	−0.117	0.168	0.451
No. of clips					
5-6	20	6.24	23.5	86.1	73.5
7-9	15	9.83	32.2	88.2	74.2
*T*		−1.807	−0.948	−1.440	0.223
P		0.080	0.353	0.159	0.825
Correction (r)	35	0.305	0.082	0.193	0.052

Abbreviations: V_mean_ = TB volume; CVS = cavity visualization score.

The data were also stratified based on the CVS, and patients with a CVS of 1 to 2 or 3 to 5 were grouped using the guideline of Landis et al. [9]. There was no significant difference in the degree of agreement between radiation oncologists (P = 0.745). The higher CVS was recorded when a given patient received conflicting CVS values. The mean CVS assigned was 2.51 ± 1.50. Overall, the assignment of CVS values by each radiation oncologist was fairly consistent. DSC_inter_ was significantly increased when the CVS was 3 to 5 compared with 1 to 2 (78.8% vs. 70.1%; P = 0.004) (Table 2). A positive correlation between the CVS and interobserver variability regarding TB delineation was thus identified.

At least 4 clips were placed at the radial extents of the cavity, and 1 clip was placed deeply [25]. When the breast tumor was anatomically deep, another clip was placed in front of the cavity. Twelve of the patients had five clips, 8 patients had six clips, 9 patients had seven clips, 4 patients had eight clips, and 2 patients had nine clips (Table 1). These patients were divided into two groups based on the surgical clip number (5 to 6 or 7 to 9). When at least 5 surgical clips were implanted in the TB, no significant differences were found in the interobserver or intraobserver delineation variability with increasing numbers of surgical clips. None of the relationship comparisons for TB volume variability and the clip number was significant (Table 2).

## DISCUSSION

TB delineation is a critical factor for EB-PBI and breast boost radiotherapy. However, the ability to strongly visualize the TB depends on the breast tissue density; the optimal number of implanted TB surgical markers; the interval from surgery to radiotherapy; tissue stranding from the surgical cavity; the target volume delineation experience; and the pre-lumpectomy setting, such as magnetic resonance imaging (MRI) and 3D ultrasound (3D-US) [7, 8, 10–13, 23, 24]. Intraobserver variation was established for each variable in the present study.

The European Organisation for Research and Treatment of Cancer (EORTC) recommends the use of a 2- to 3-mm CT slice thickness because this slice thickness generates high-resolution digitally reconstructed radiographs, which facilitates accurate tumor delineation for the planning and delivery of high-dose, high-precision radiotherapy for lung cancer (grade of recommendation/description: 1B) [26]. Additionally, recent research has found that thin-slice 3D reconstruction images more clearly reveal the morphological characteristics of tumors and breast tissues and the margins of different tissues in each slice [27]. In the present study, variation of the reconstructed 4D-CT slice thickness did not produce inter(intra)observer variability among radiation oncologists in TB delineation in patients with early breast cancer who had undergone BCS. However, the tumor shapes that were obtained using the two reconstruction methods were basically similar after 3D reconstruction. Therefore, thin-slice images and differences in the reconstruction slice thickness of CT images did not improve the accuracy of surgical cavity delineation for patients with surgical clips that were placed during lumpectomy.

4D-CT provides information on the motion of mediastinal structures, which improves the delineation of target volume. Our prophase research has demonstrated that intrafraction target displacement during free breathing was limited, and the influence of intrafraction target displacement on the dose distribution is not significant during irradiation [19–21, 28–30]. However, no prior studies about translation of 4D-CT technical advances into clinical advantages, such as tumor control or survival, have been performed. Therefore, the integration of 4D-CT into the target delineation process was rational. A recent study by Cover et al. [31] found that the EE position of a tumor was maintained approximately 50% of the time and reduced the uncertainty in tumor location resulting from respiration. Therefore, all delineations were established at the EE phase in the present study. The ability to visualize the TB strongly depends on the breast tissue density [8]. Age, tumor size, the interval from surgery to radiotherapy, and the pathological resection volume are not related to breast density values. The present study assessed the correlation between both delineation variability and the DSC and the TB volume, and we found that the interobserver DSC for TB determination increased approximately 7% in women with a larger TB size and that the interobserver variability in delineation (Δ_inter_) decreased from 37.0% to 15.6%. Landis et al. [9] recently demonstrated that the average shift in the center of mass was significantly smaller when the pathological diameter was > 4 cm using multiple linear regression. Therefore, in the current study, the TB boundaries for patients with a smaller TB exhibited low delineation concordance, despite the placement of surgical clips within the TB. CT imaging with MRI, 3D-US or PET/CT would benefit patients with a low-volume TB, which is associated with low simulation and TB delineation variability [13, 32, 33].

The TB volume constantly changes after surgery, and the CVS gradually declines. Therefore, a good grasp of the appropriate timing for simulation scanning is needed in this case. A lower Δ_inter_ or higher DSC value indicates better convergence among volumes and higher similarity among the compared contours. In the present study, patients with more than five surgical markers exhibited an improvement in TB delineation, with CVS scores of 3 to 5, and the interobserver variability in TB delineation was reduced by 11.7% (from 32.2% to 20.6%). Kader et al. [34] and Lewis et al. [35] suggested that the optimal time to obtain the planning CT scan for radiotherapy is within 8 to 9 weeks after surgery (and not more than 14 weeks afterward) to ensure that the seroma remain adequately defined in most patients. Given that the TB volume changes dramatically during this stage, combination of the CT-based seroma with surgical clips should provide a suitable guide for TB target volume definition. An inclusive delineation using the seroma and surgical clips should specifically be performed when a spatial mismatch exists among the TB volumes delineated based on the seroma and surgical clips.

The selection of the appropriate time from lumpectomy to CT simulation scanning and the use of a sufficient number of surgical clips must be considered to minimize errors in defining the TB [7, 8, 11, 25]. Clips implanted in surgical cavity walls provide additional localization information, but detachment or gathering of surgical clips appears over time after surgery and with increasing gradual mobility. Therefore, surgical clips do not completely represent the TB border. Many surgeons recommend the placement of 7 to 10 surgical markers at the time of surgery, although some prefer not to place clips. Ippolito et al. [5] investigated the percentage of intersection volume among clip-based clinical target volumes (CTVs) for PBI delineated on postoperative CT scans and the initial tumor location on preoperative CT scans and noted that surgical clips were essential and that the use of six or more clips led to inaccurate TB definition. However, pre- and postoperative CT image co-registration has limitations. First, limited soft tissue contrast on CT may make it difficult to visualize all breast tumors. Second, breasts are distorted after surgery. Interobserver variability was minimal for TB delineation based on seroma when the CVS was 3 to 5 points and when at least 5 surgical clips were placed during BCS in our preliminary experiments [28]. The present study demonstrated that the placement of more than 5 to 6 surgical clips at the time of lumpectomy did not improve the DSC, with less interobserver variability in TB delineation after BCS. However, a key limitation of our study was that it was only comparative, and we could not ensure the absolute error in determining the TB.

Our study should be interpreted while keeping two limitations in mind. First, our study was comparative only, and the absolute error in determining the TB cannot be determined because the exact post-lumpectomy cavity extent is not known. A multidisciplinary approach to boost the breast TB would be useful, such as injection of a contrast agent into the cavity, 3D-US or PET/CT use or MRI simulation and use in contouring the TB. Second, the sample size of this study might lead to uncertainty, as the possibility that the results are not relevant to a larger sample size cannot be excluded.

## MATERIALS AND METHODS

### Patient selection and instruction

All patients had undergone tumorectomy and sentinel node and/or lymph node dissection for early breast cancer in our department from June 2014 to March 2015, with five or more surgical clips placed in the TB during surgery. These recruited patients were suitable for postoperative treatment with EB-PBI or the simultaneous integrated boost (SIB) technique. Patients with restricted arm movements after surgery or poor pulmonary function were excluded. Written informed consent was obtained from all patients, and the institutional research ethics board of Shandong Cancer Hospital approved this study.

### 4D-CT data acquisition

Patients were immobilized in a supine position on a breast board, with both arms above their head and positioned on an arm support device during the simulation. A knee support was placed under the knees to fix the patients' position and improve their comfort.

Free-breathing 4D-CT scans were acquired using a 16-slice CT scanner (Philips Medical Systems, Inc., Cleveland, OH, USA). Respiratory signals were recorded using the Varian Real-Time Position Management (RPM) System (Varian Medical Systems, Palo Alto, CA, USA) by measuring the displacement of infrared markers placed on the epigastric region of the patient's abdomen. GE Advantage 4D software (GE Healthcare, Waukesha, WI, USA) was used to sort the reconstructed 4D-CT images into ten respiratory phases, labeled as 0 to 90% based on triggered signals. Phase 0% denoted the maximum end inspiration (EI), and phase 50% denoted the maximum end expiration (EE). The 4D-CT images were transferred to the Eclipse Treatment Planning System (TPS) (Eclipse 8.6, Varian Medical Systems, Palo Alto, CA, USA) for structure delineation.

### TB contouring methods

First, four radiation oncologists delineated the TB contours on the EE phase for each patient. All four radiation oncologists were given the following criterion for TB delineation: the TB boundaries were defined using a combination of breast tissue changes that were apparent on CT simulation, pathological and radiographic information, fluid collection within the TB, and the number of surgical clips. Second, one experienced radiation oncologist was asked to contour the TB an additional three times to test intraobserver variability. Intraobserver variability and interobserver variability in TB contouring were defined as Δ_intra_, and Δ_inter_, respectively. Δ_inter_ (Δ_intra_) was the ratio of the inter(intra)observer TB volume differences in the delineation to the average volume.

### TB volume analysis

The Dice similarity coefficient (DSC) was used to determine the extent of spatial overlap between two regions of interest, ranging between 0 (no overlap) and 1 (perfect overlap) [36]. The DSC was defined as

DSC=(2|A∩B|)/(|A∩B|+|A∪B|)×100%

DSC_intra_ was the percentage ratio for intraobserver variability in TB delineation, and DSC_inter_ was the percentage ratio for interobserver variability in TB delineation.

### Statistical analysis

Statistical analysis was performed using the SPSS statistical analysis software package (SPSS Inc., Chicago, IL, USA). T-tests or one-way analysis of variance (ANOVA) was performed to determine the statistical significance of differences in the variability of TB delineation among the different groups, depending on the normality of the data. The Pearson correlation test was also used to examine the relationship between the target variation and influencing factors. A P-value < 0.05 was considered statistically significant. All significance levels were two sided.

## CONCLUSIONS

The present study demonstrated that the TB volume and CVS were the primary influencing factors among different observers when 5 or more surgical clips were placed at the TB. However, using more than 6 surgical clips within the TB did not increase the accuracy of TB delineation in 3D EB-PBI or a TB boost for WBI. We note the importance of surgical clips in providing TB delineation and recommend the placement of 5 to 6 surgical clips to allow adequate TB localization following BCS. This approach is also preferable for the purposes of TB delineation. The TB field depends on the surgeon's choice and relates to tumor size. Breast tissue is nearly always remodeled during breast cancer surgery, with either full-thickness closure of the lumpectomy or superficial closure. Therefore, TB delineation using the seroma or clips may result in geographical misses of the primary tumor after surgery. Further evaluations are thus needed to clearly define the role of the combination of the pre-lumpectomy and postoperative settings in TB contouring.
